# Crafting Engagement Before Entering the Profession: Pre-Service EFL Teachers’ Experiences of Proactivity and Flow

**DOI:** 10.3390/bs16050758

**Published:** 2026-05-12

**Authors:** Feyza Nur Ekizer, Aydan Irgatoğlu

**Affiliations:** 1 School of Foreign Languages, Necmettin Erbakan University, Konya 42090, Türkiye; fekizer@erbakan.edu.tr; 2 School of Foreign Languages, Ankara Hacı Bayram Veli University, Ankara 06500, Türkiye

**Keywords:** proactive personality, flow, work engagement, job crafting, pre-service EFL teachers

## Abstract

This study examines how pre-service English teachers in Turkey experience the dimensions of proactive personality, job crafting, flow, and work engagement while making sense of their professional lives. In the phenomenological design research, semi-structured interviews were conducted with 13 teacher candidates selected through purposive sampling. Qualitative data were transcribed and subsequently analyzed using MAXQDA 2022 qualitative data analysis software and high inter-coder reliability was found. The findings were grouped under four themes. Under the heading of proactive personality, solution-focus, patience and communication, seeking support, and continuous development came to the fore. In the flow experience, open feedback from students, activities that made learning enjoyable, changes in the perception of time, and full participation in the natural flow of the lesson stood out. Professional craftsmanship manifested itself through strategies such as establishing trust-based relationships with students, colleagues, and parents, adapting methods, self-care, and time management. Dedication to work was defined through passion for the profession and the vitality provided by working with different student profiles. The results showed that teacher candidates demonstrated resilience-oriented professional strategies by combining individual initiative, social support, and continuous development tendencies. Furthermore, student-centered feedback and community-based relationships are understood to strengthen flow experiences and nurture dedication to work. The study points to the importance of supporting proactive tendencies in teacher training and designing learning environments conducive to flow.

## 1. Introduction

In the dynamic landscape of language education, teachers occupy a central role in shaping instructional quality and student learning outcomes through their everyday pedagogical decisions and interactions (Borg, 2024; Farrell, 2018; Gordon, 2006; Y. Han & Wang, 2021; Richards, 2015). Beyond content expertise, effective teaching requires sustained psychological investment, adaptive professional behaviors, and the capacity to respond proactively to evolving instructional demands. Recent research has increasingly emphasized that what distinguishes impactful educators is not only what they know, but also who they are, including their personal dispositions, experiential states, and proactive work behaviors that jointly shape professional engagement (Derakhshan, 2022; Dewaele et al., 2018).

From this perspective, teacher effectiveness cannot be fully understood without considering how internal psychological resources interact with behavioral strategies that allow teachers to shape their work environments. Teachers are not passive recipients of institutional conditions; rather, they actively modify their tasks, relationships, and professional roles in ways that influence both their motivation and well-being. This dynamic interplay between dispositional traits, proactive work behaviors, and optimal experiential states forms the foundation of sustained work engagement in language education (Kim et al., 2019).

Within this integrated framework, several internal psychological and behavioral resources have been identified as central to sustaining teacher motivation and instructional quality, most notably work engagement, flow, proactive personality, and job crafting (Amini Faskhodi & Siyyari, 2018; Bakker et al., 2012; Fu et al., 2024; Greenier et al., 2021). Work engagement, characterized by vigor, dedication, and absorption, represents a positive and fulfilling work-related state that reflects teachers sustained motivational investment in their profession (Schaufeli et al., 2002). Rather than functioning as a static outcome, engagement is increasingly conceptualized as a dynamic state shaped by teachers’ dispositions, proactive behaviors, and lived teaching experiences.

In the teaching profession, high levels of engagement are associated with enhanced instructional effectiveness, resilience, and professional commitment (Cardwell, 2011; Derakhshan et al., 2022; Roth et al., 2007). Conversely, persistently low engagement may undermine instructional quality even when teachers remain in the profession, highlighting the importance of understanding the processes that sustain engagement over time (Darling-Hammond & Youngs, 2002). Accordingly, recent research has shifted attention toward identifying the psychological and behavioral mechanisms through which engagement is cultivated, particularly in demanding EFL contexts (Greenier et al., 2021; Hong, 2010; Mérida-López et al., 2020).

One emerging factor contributing to teacher engagement is the experience of flow, defined as a state of deep, intrinsically motivated involvement in an activity. Flow is characterized by three core dimensions: deep concentration, enjoyment, and intrinsic motivation (Bakker & van Woerkom, 2017; Ryan & Deci, 2000). In teaching contexts, flow functions as a valuable psychological resource, enabling educators to navigate professional challenges with heightened focus and enthusiasm. Although both flow and proactive personality are situated within the broader framework of positive psychology (Dewaele et al., 2019; Y. Wang et al., 2022; Irgatoğlu & Yurttadur, 2026), their potential interactive effects on work engagement remain largely unexplored in EFL teaching settings. Importantly, flow is not merely an isolated experiential state; rather, it often emerges through teachers’ proactive efforts to align instructional challenges with their skills and interests, indicating a close connection between flow experiences, adaptive work behaviors, and sustained engagement.

Another psychological factor that may play a pivotal role in teacher engagement is proactive personality, defined as an individual’s tendency to take initiative and instigate positive changes within their environment (Parker & Wang, 2015). Proactive individuals are typically characterized by resilience in the face of constraints, innovative thinking, and a strong motivation to seek and capitalize on opportunities (Chai et al., 2023). Empirical research has demonstrated a robust relationship between proactive personality and work engagement across diverse professional contexts (Bell & Staw, 1990). In educational settings, proactive teachers may be particularly inclined to initiate instructional improvements and adapt their teaching practices in response to evolving demands (Bakker et al., 2012). However, research examining this relationship within EFL-specific contexts remains limited, particularly among pre-service teachers.

Although prior research has investigated various individual antecedents of teacher work engagement, including emotional regulation, psychological well-being, and instructional beliefs (Caires et al., 2012; Greenier et al., 2021; Mérida-López et al., 2020; Derakhshan et al., 2022), these constructs have typically been examined in isolation rather than as part of a dynamic system of interrelated psychological and behavioral processes. Moreover, the behavioral processes through which proactive dispositions translate into optimal experiential states and sustained engagement remain insufficiently explored. Specifically, little is known about how proactive personality shapes teachers’ job crafting practices, how these practices facilitate flow experiences, and how such interrelated processes jointly sustain work engagement over time.

This gap is especially evident in the context of Türkiye, where empirical research on teacher engagement and its underlying psychological and behavioral mechanisms remains limited. Addressing this gap is critical for advancing a more integrative understanding of teacher motivation that accounts for dispositional, behavioral, and experiential dimensions. To this end, the present qualitative phenomenological study investigates how proactive personality, job crafting, flow, and work engagement interact to sustain work engagement among pre-service EFL teachers in Türkiye. Guided by an integrative framework, the study examines the sequential interplay of proactive personality as a dispositional resource, job crafting as a behavioral mechanism, flow as an experiential state, and work engagement as a motivational outcome.

## 2. Review of Literature

### 2.1. Teacher Work Engagement as a Core Outcome

Teaching is widely recognized as a demanding profession, often accompanied by high levels of occupational stress and an increased risk of burnout. Despite these challenges, many educators remain psychologically invested in their work, a phenomenon conceptualized as work engagement (Bao et al., 2021; Schaufeli et al., 2009; Xing, 2022). Work engagement is defined as a positive, fulfilling, work-related mental state characterized by vigor, dedication, and absorption (Schaufeli et al., 2002). Vigor refers to high levels of energy and resilience, dedication reflects a sense of significance and enthusiasm, and absorption denotes deep immersion in work-related activities (Hakanen et al., 2006; Lu et al., 2018).

Within educational contexts, engaged teachers are more likely to demonstrate persistence, enthusiasm, and professional commitment, thereby contributing to positive instructional outcomes and broader school development (Bakker & Bal, 2010). Empirical studies further indicate that work engagement functions not only as an indicator of well-being but also as a motivational resource that supports proactive behavior, resilience, and organizational commitment (Hakanen et al., 2006; Salanova & Schaufeli, 2008).

In the Turkish context, however, research on teacher work engagement remains limited. Existing studies report moderate levels of engagement among teachers (Azari Noughabi et al., 2022; Gün, 2017; Güçlü et al., 2014), raising concerns given the well-established association between teacher engagement and student achievement (Darling-Hammond & Youngs, 2002). As Klassen et al. (2012) argue, teacher engagement is essential not only for instructional quality but also for reducing burnout and fostering active participation in school improvement initiatives. From this perspective, work engagement is best understood not merely as an individual outcome, but as a dynamic motivational state shaped by teachers’ dispositional traits, proactive behaviors, and experiential involvement in their work.

### 2.2. Flow as an Experiential Pathway to Engagement

Recent scholarship grounded in positive psychology has emphasized the role of optimal psychological experiences in sustaining motivation and professional vitality (Hultell & Gustavsson, 2011; Klassen et al., 2014; Klusmann et al., 2008). One such experience is flow, defined as a state of deep absorption, enjoyment, and intrinsic motivation that arises when individuals perceive a balance between task demands and personal skills (Csikszentmihalyi, 2003). In professional contexts, flow is characterized by intense concentration, a sense of control, and intrinsic satisfaction derived from task engagement (Bakker, 2005, 2008; T. Wang et al., 2023).

Conceptually, flow aligns closely with the absorption dimension of work engagement, suggesting that frequent flow experiences may function as an experiential mechanism through which engagement is maintained (Hakanen et al., 2006; Yurttadur, 2014). Empirical evidence further indicates that flow enhances resilience, persistence, and performance, particularly in demanding work environments (Bakker, 2008; Shakki, 2022).

In educational contexts, teachers may experience flow when instructional challenges align with their pedagogical skills and when classroom interactions are perceived as meaningful and rewarding. Qualitative research suggests that EFL teachers frequently report flow experiences during teaching activities, especially when student engagement is high (Tardy & Snyder, 2004). Despite its theoretical relevance, empirical research examining flow among EFL teachers, particularly in relation to work engagement, remains limited. These findings indicate that flow may function as an experiential mechanism through which teachers’ proactive adjustments to their work translate into sustained engagement, especially within demanding EFL teaching contexts.

### 2.3. Job Crafting as a Behavioral Link Between Personality and Engagement

While flow captures the experiential dimension of teaching, job crafting represents a behavioral process through which teachers actively shape their work to enhance meaning and engagement. Job crafting refers to employees’ proactive efforts to modify their tasks, relationships, or cognitive perceptions of their work in order to increase its meaningfulness (Wrzesniewski & Dutton, 2001). Drawing on the Job Demands Resources model (Demerouti et al., 2001), job crafting enables individuals to mobilize job resources such as autonomy, feedback, and social support, which foster engagement and well-being.

Job crafting is commonly conceptualized in three forms: task crafting, relational crafting, and cognitive crafting. Through these strategies, teachers may redesign instructional activities, alter interaction patterns with students and colleagues, or reinterpret their professional roles in more meaningful ways. Research has shown that such proactive adjustments enhance job satisfaction, motivation, and performance (Grant, 2007; Hinds et al., 2015; Rosso et al., 2010).

Importantly, job crafting has been identified as a key antecedent of work engagement, as it allows individuals to align their work environments with personal strengths and values (Bakker et al., 2012; Yurttadur, 2015). In educational settings, job crafting may serve as a mechanism through which teachers sustain engagement despite high job demands, particularly when institutional support is limited. Accordingly, job crafting can be conceptualized as a behavioral bridge that connects proactive personality traits with flow experiences and, ultimately, higher levels of work engagement.

### 2.4. Proactive Personality as an Individual Resource

At the dispositional level, proactive personality has been identified as a critical personal resource that enables teachers to initiate job crafting behaviors, experience flow, and sustain work engagement (Bateman & Crant, 1993). Proactive individuals are characterized by initiative-taking, future-oriented thinking, and a willingness to act upon opportunities for improvement (Fuller et al., 2012; Hakanen & Schaufeli, 2012). Such traits are particularly valuable in educational contexts, where teachers frequently encounter uncertainty, workload pressures, and evolving pedagogical demands.

Research suggests that proactive individuals are more likely to engage in job crafting behaviors, seek feedback, and adopt adaptive coping strategies (Ng & Feldman, 2013; Straud et al., 2015). In this way, proactive personality may indirectly contribute to work engagement by enabling teachers to restructure their work and create conditions conducive to flow experiences. Moreover, proactive teachers tend to demonstrate stronger emotional regulation and resilience, allowing them to transform challenges into opportunities for professional growth (Klassen & Tze, 2014; Şahin, 2006; Skaalvik & Skaalvik, 2015).

Despite its relevance, the role of proactive personality in shaping EFL teachers’ engagement and flow experiences remains underexplored. While previous studies have examined related constructs such as teacher immunity, creativity, and psychological well-being (Derakhshan et al., 2022; Kong et al., 2021; Sadeghi & Khezrlou, 2016), few have explicitly addressed how proactive dispositions interact with experiential and behavioral factors to sustain engagement.

Taken together, these strands of research point to a coherent process in which proactive personality influences how teachers shape their work, experience optimal engagement states, and maintain long-term motivational investment. The literature suggests that work engagement functions as a central motivational outcome, flow represents an optimal experiential state, job crafting serves as a behavioral strategy, and proactive personality acts as an underlying personal resource. These constructs are theoretically interconnected: proactive teachers are more likely to engage in job crafting, which enhances the meaningfulness of teaching and increases opportunities for flow, thereby sustaining high levels of work engagement.

However, existing research has largely examined these constructs in isolation, particularly within EFL contexts. There remains a clear need for integrative research that captures how dispositional, behavioral, and experiential factors jointly contribute to teachers’ professional vitality. Addressing this gap, the present study investigates the interrelationships among proactive personality, flow, job crafting, and work engagement among pre-service EFL teachers, offering a more holistic understanding of teacher motivation and resilience in language education. Taken together, the reviewed literature highlights the interconnected roles of proactive personality, job crafting, flow, and work engagement in shaping teachers’ professional experiences. However, these constructs have predominantly been examined in isolation, with limited attention to how they interact as part of a dynamic and integrative process, particularly within EFL contexts and among pre-service teachers. Building on this gap, the present study adopts a qualitative phenomenological approach to explore how these dimensions are experienced and interrelated in the context of teacher preparation in Türkiye. Drawing on this integrative framework, the present study is guided by the following research questions:

- How do pre-service EFL teachers describe their proactive personality traits in relation to anticipating and managing professional challenges?
- How do proactive personality traits manifest in pre-service EFL teachers’ job crafting practices?
- How do job crafting practices facilitate flow experiences during teaching-related activities?
- How do proactive personality, job crafting, and flow interact to sustain pre-service EFL teachers’ work engagement?

## 3. Materials and Methods

### 3.1. Design

In the present work, a qualitative phenomenological research design was used in order to investigate the lived experiences of pre-service English as a Foreign Language (EFL) teachers in Turkey in relation to their proactive personality, flow experiences, job crafting strategies, and work engagement. Within the scope of this investigation, flow experiences are operationalized as states of focused attention and cognitive absorption that preservice teachers may encounter during specific pedagogical activities, such as lesson preparation, material design, or active instructional delivery. It was selected because phenomenological inquiry enables the deeper comprehension of the nature of the subjective experience and perception of the participants within the context of their professional development (Creswell, 2018). The design was geared towards uncovering the meanings that the participants attribute to challenges, motivations and adaptive behaviours in teaching that is consistent with the interpretive paradigm that places emphasis on the voices of the participants and on the realities of the context.

### 3.2. The Participants

The purposive sampling method was used to identify participants who would be pertinent to the phenomenological inquiry because of their pertinent attributes (Dong & Xu, 2022; Patton, 2015). Specifically, the sample comprised pre-service EFL teachers who are currently undertaking teacher education at Turkish universities and who have adequate practical experience to comment intelligibly on the teaching realities. Regarding the eligibility of participants, candidates who were in the final year of bachelor’s degree in EFL education were given a priority in the selection process in order to guarantee that there is enough practicum experience. Furthermore, in order to guarantee the richness of the data and the professional relevance, the research targeted persons who explicitly indicated a clear intention to pursue a teaching career after graduation, and who volunteered to provide detailed reflections on their professional motivation and adjustment (Table 1).

The research involved 13 respondents (labeled as P1 through P13 in the findings) who covered both sexes and different parts of Türkiye to express different opinions. All participants were enrolled in the EFL teacher education program at the same institution. However, they were purposefully selected among students originating from various geographical regions across Türkiye. This purposeful selection within the single institutional context aimed to capture a diverse range of cultural backgrounds and lived experiences. The sampling was conducted until saturation of data, meaning the point when it was not possible to gain new themes and insights in the process of conducting additional interviews, was achieved (Saunders et al., 2018). Saturation was operationalized not just in terms of the number of interviews, but in terms of redundancy of emerging codes. Analysis showed that no new distinct themes or codes were produced after the 11th interview. Consequently, two additional interviews were undertaken to confirm this saturation and the stability of the data set. Ethical issues were observed including informed consent, anonymity and allowing the participants to withdraw at any time they desired. The institutional ethics review board gave approval to the study.

### 3.3. Data Collection Tools and Procedure

The study was conducted during the [2024–2025] academic year. Data were collected through semi-structured individual interviews, which are well-suited for phenomenological studies as they facilitate open-ended exploration while maintaining focus (Moustakas, 1994). Interviews were conducted face-to-face or via video conferencing (e.g., Zoom) in Turkish to ensure participants’ comfort and natural expression, lasting approximately 30–45 min each. The interview protocol consisted of 10 open-ended questions designed to elicit detailed descriptions of participants’ points of view. Questions were pilot-tested with two non-participant pre-service teachers to refine clarity and flow, resulting in minor adjustments for better probing. To enhance methodological transparency, the questions used as the basis for the interviews are listed below:

1. What does it mean to be a teacher?
2. What do you think motivates a teacher the most?
3. What will give you the most joy in your profession?
4. When you become a teacher, what role will students, colleagues, and parents play in your life?
5. What challenges do teachers face in their careers, and how can they overcome them?
6. As a future teacher, how do you think you will overcome difficulties?
7. What factors provide resilience to a teacher?
8. When does a teacher perform their job at their best?
9. What keeps a teacher in the profession?
10. What is the role of emotions in a teacher’s professional life? How do teachers manage their emotions? How will you handle them?

### 3.4. Data Analysis

Qualitative data were transcribed and subsequently analyzed using MAXQDA 2022 qualitative data analysis software by following Braun and Clarke’s (2006) six step process of thematic analysis: familiarization with data, generating initial codes, searching for themes, reviewing themes, defining and naming themes and writing up the report.

To ensure the trustworthiness of the study, strategies based on Lincoln and Guba’s (1985) criteria, i.e., credibility, transferability, dependability and confirmability were implemented (Nowell et al., 2017).

- **Credibility** was ensured by member-checking, whereby participants examined their transcripts for accuracy, and peer debriefing, whereby the research team debated emerging codes to reduce individual bias.
- **Dependability** was ensured by keeping an in-depth audit trail of all the methodological decisions taken during the project.
- **Confirmability** was addressed by researchers engaging in “bracketing” to put aside their own assumptions and focus solely on the experiences of the participants.

Two researchers independently coded each transcript and mathematically calculated inter-coder reliability rate >80% using the formula: (number of agreed codes/total number of generated codes) × 100. This level of agreement shows strong consistency and credibility of the thematic analysis findings.

## 4. Results

Below, findings of the thematic analysis are presented.

As shown in Table 2, the qualitative findings relating to proactive personality are organized under three major themes: initiative in problem solving, seeking and using support, and continuous self-improvement and self-advocacy. The analysis of these themes shows that proactivity is not only perceived by pre-service teachers as a personality trait, but as a dynamic set of professional strategies.

The analysis of the narratives of the participants indicates that the pre-service teachers take a “constructive interventionist” role when confronted with educational problems. Rather than passively accepting obstacles, they cognitively reframe problems as tasks to be accomplished with specific strategies. This finding suggests that proactivity in the teaching context is highly related to resilience and emotional regulation. Participants stressed that it is not only important to find a quick fix to the problem, but rather to approach problem solving with a deliberate process of communication and patience. This proactive approach changes the role of the teacher from one of a victim of circumstance to that of an agent of change. A representative statement to explain this solution-oriented way of thinking and strategic use of patience is as follows:

*“I believe that I can handle problems through communication and understanding. However, if I face a more serious issue, I think patience is the key to overcoming it.”*

(P9)

*“I will approach challenges with a solution-oriented mindset…”*

(P1)

Although proactivity is mostly seen as an individual trait, the results of this research point to the social aspect of proactivity. The results of this analysis show that “resource mobilization”—the ability to identify and utilize social support networks—is a critical component of the proactive behavior of the participants. Teacher candidates do not see asking for help as an indication of weakness, quite the opposite, they strategically seek collaboration with colleagues and parents to increase their ability to solve problems. This would imply that professional resilience is built not in isolation but collectively. The following excerpt illustrates how participants combine the solution-focused mindset with the proactive seeking of collegial support:

*“To handle challenges…approaching issues with a solution-focused mindset … and seeking support from colleagues when needed will be essential strategies for me.”*

(P11)

*“I will … seek support from colleagues or parents when needed …”*

(P2)

The last theme suggests that the participants consider proactivity as a long-term investment in their professional identity. The data implies that for these teacher candidates to be proactive means that they have a double responsibility of structuring their work such that they avoid problems before they happen (prevention) and they continuously up-grade their pedagogical skills (development). Furthermore, “self-advocacy” (knowing one’s professional rights) became an important proactive means to maneuver within institutionalized constraints. This is a mature view of the profession, in which growth is viewed as a journey, a continuous life-long learning rather than a destination. One participant expressed this multidimensional structured planning and rights awareness as follows:

*“First and foremost, I will ensure that my lesson plans are well-structured to prevent any disruptions. Additionally, I will be aware of my rights as a teacher to handle any challenges effectively.”*

(P6)

Similarly, another participant highlighted the aspect of continuous growth:

*“To overcome challenges … I aim to continuously improve myself. By reading, learning, and gaining experience …”*

(P10)

As illustrated in Table 3, the phenomenon of flow among pre-service teachers can be split into four sub-themes that drive each other’s development, which are the following: Unambiguous Feedback, Autotelic Experience, Time Transformation, and Awareness of Actions Merged. It is suggested that these dimensions are not strictly dependent on one another for their initial manifestation; rather, they appear to interact in a complementary manner. The progression of one sub-theme may facilitate the development of the others, indicating a mutually supportive, though not absolutely reliant, dynamic. The interpretation of the results of this analysis is that for these teachers, flow is not just a passing emotion but is a structural state of high cognitive and pedagogical performance.

The findings suggest that for pre-service teachers the teaching process involves a high degree of “pedagogical verification loop.” Unlike professions where feedback is received later on, participants named the immediate, observable reactions of students as the main trigger for the flow state. The analysis suggests that unambiguous feedback is a mechanism of validation; when a student “gets it,” it validates the teacher’s efficacy in real-time, and prevents doubt and maintains the focus. Far from waiting for the exam results, for teachers these micro-moments of clarity are where they get their best experience. One participant has described this unique moment of realization as being a powerful source of joy:

*“For example, if I had a student who suddenly said, ‘I got it!’ and I saw that moment of realization in their eyes, that would bring me joy.”*

(P8)

Another participant emphasized that this feedback extends beyond the classroom, viewing the real-life application of knowledge as the ultimate confirmation of their instructional impact:

*“I believe one of the most motivating things for a teacher is…witnessing students implementing what they have learned in their lives…”*

(P1)

The theme of autotelic experience reveals that the participants see teaching as an activity that has its own intrinsic reward system. The data calls which is known for its a crucial difference between “labor” which is motivated by external incentives (like salary) and “craft” which is motivated by internal passion. Participants explicitly located their teaching (the design of creative activities and the process of guiding students) as a source of fulfillment overriding financial gain. This intrinsic orientation seems to be a protective factor against burnout where the effortful nature of teaching is pleasant rather than exhausting. This sentiment was well expressed by one participant who compared financial motivation with professional passion:

*“Earning money and gaining financial independence can motivate a teacher at some point but teaching children with passion gives more motivation than money.”*

(P4)

Similarly, the joy derived from the act of pedagogical design itself was highlighted as a key driver of this autotelic state:

*“… Organizing creative activities and games in the classroom to make learning more enjoyable will bring me great joy.”*

(P10)

The last two sub-themes, time-transformation and action-awareness merging, are the phenomenological “symptoms” of deep absorption in teaching. The analysis suggests that it is the combination of high skill and high challenge that causes the pre-service teacher’s self-consciousness to dissolve. “Time transformation” means losing a track of time. Simultaneously, “action-awareness merging” refers to a state of fluency in which the teacher no longer “thinks about” teaching, but just “is” teaching; the gap between the plan and the execution disappears. This state of total immersion was described in the following way according to participants:

*“The moments when a teacher immerses themselves in a topic, completely forgetting about time … are also highly motivating.”*

(P8)

*“… Being immersed in the natural flow of lessons allows teachers to engage seamlessly in activities and ensure smooth progression in the classroom.”*

(P13)

Table 4 summarizes the important dimensions of job crafting that were identified in the analysis. The findings imply that pre-service teachers do not see their future roles as fixed descriptions given to them by the ministry or school administration, but rather re-design the physical, social, and cognitive boundaries of their work to fit with their personal strengths and professional needs. The four sub-themes, namely, relational crafting, task crafting, decreasing hindering job-demands, and cognitive crafting, show this active shaping process.

The analysis of relational crafting shows that the participants deliberately attempt to enlarge the social boundaries of their job. Rather than thinking of teaching as an insular activity from the four walls of a classroom, they want to create a “support ecosystem” with students’ needs, colleagues, and parents. This strategy turns the profession from an individual activity to a communal one. Participants stressed that building deep connections of trust is not only a social preference, it is a professional necessity to improve the climate in the schools. One of the participants expressed the significance of this triangular solidarity (colleagues-parents-support) as follows:

*“As for colleagues, they can form a community that supports development through solidarity and sharing. Regarding parents, I believe establishing a trust-based and supportive communication is crucial.”*

(P8)

Another participant highlighted the specific intent to deepen the teacher-student bond over time:

*“Over time, I hope to develop deeper relationships with students…”*

(P6)

Under the theme of task crafting, the findings show teacher candidates to display “pedagogical agility.” When confronted with student disengagement or routine, they will not continue with ineffective methods, but will alter the nature of the work. This means adopting new teaching techniques or changing the extent of their work in order to make the work interesting for both themselves and their students. This proactive modification is an antidote to monotony and student passivity. A good example of this adaptability to student need in the classroom was given by one participant:

*“When students are not engaged … The best way to overcome this is by changing teaching methods …”*

(P5)

Moreover, this crafting extends to professional development, viewing continuous learning not as an obligation but as a tool to refine their craft:

*“… Continuously developing oneself, and exploring new teaching methods are essential strategies.”*

(P13)

The theme of decreasing job demands is indicative of a protective mechanism used by the participants. The analysis suggests that pre-service teachers are keenly aware of the hazards of burnout, emotional exhaustion and overloading due to bureaucracy. Consequently, they engage in job crafting in terms of mentally or physically minimizing these draining aspects. This is not an avoidance of responsibility, but a strategic “resource conservation” in order to ensure their long-term well-being and effectiveness. Participants described self-care and boundary-setting as necessary professional skills:

*“Emotional exhaustion, curriculum pressure … To overcome these, teachers should develop their own strategies … and take time for themselves.”*

(P8)

*“… Working in a supportive school environment … and implementing self-care strategies will allow teachers to remain strong and overcome bureaucratic obstacles.”*

(P11)

Finally, cognitive crafting emerged as a psychological strategy in which the participants change their perception of the job rather than the job itself. The data reveal that in the case of physical or structural changes not being possible, teachers “reframe” challenges (such as difficult students or systemic limits) as opportunities for growth or tests of patience. This mental shift enables them to maintain a positive professional identity in spite of external stressors. One participant captured this rational reframing process very well:

*“Teachers face many challenges … they must remain patient and rational … by cultivating patience, many challenges can be managed effectively.”*

(P7)

Table 5 shows the thematic structure of work engagement, which has two different, yet complementary, sub-themes; dedication and vigor. The results indicate that for pre-service teachers, engagement is not an immutable characteristic but an active and persistent state of mind that is defined by deep emotional commitment and high energy.

The theme of dedication shows that the commitment of the participants to the profession is rooted in a deep sense of “vocational significance.” It is not just about holding a job, but the value in the contribution of their lives to the lives of the students. The analysis suggests a two-way mechanism: teachers maintain passion and effort into their teaching and, in turn, look for the validation of “making a difference.” This perceived impact serves as a sustainment factor for their career longevity. One participant stated this passion as the driving force:

*“Passion for education and a genuine desire to make a difference in students’ lives play a significant role.”*

(P3)

Furthermore, the information calls attention to the fact that this commitment is future-oriented. The perception that their work is meaningful gives them a buffer against the risk of attrition and encourages them to remain in the profession for the long term. As one participant commented, seeing the actual results of their labors helps them become more committed:

*“When a teacher feels that their work is valuable and sees the impact of their efforts, they are more likely to continue their career for many years.”*

(P10)

The sub-theme of vigor emphasizes participant physical and mental energy in work. Interestingly, the analysis suggests that pre-service teachers are energized by challenges in the profession, while not being exhausted by them. The “ever-changing student profile” is seen not as a stressor, but as a dynamic stimulus, which forces the teacher to be flexible and alert. This is a way of thinking that the complexity of the classroom becomes the professional liveliness which keeps the classroom from stagnating. One startling example of this way of thinking, of where difficult students were seen as a testing ground for skills, was given by P4:

*“The ever-changing student profile gives vitality to the teacher. The teacher tests his/her own teaching skills against the difficult student.”*

(P4)

## 5. Discussion

The findings of this study suggest that teacher candidates rely on four positive psychological and behavioral dimensions (i.e., proactive personality, flow, job crafting, and work engagement) in making sense of their professional lives. While the results seem to be broadly consistent with earlier research (e.g., Gordon, 2006; Guetterman et al., 2015; Schaufeli et al., 2002; Wrzesniewski & Dutton, 2001; Csikszentmihalyi, 2003; Parker & Wang, 2015), they have unique nuances, especially in the EFL context, where the dimensions of “student-generated open feedback” and “relational crafting” are prominent. Rather than working in isolation, the analysis suggests that these constructs work as an interrelated developmental process for pre-service teachers.

Regarding the first dimension, the results obtained on proactive personality (Table 2) reveal that tendencies towards initiative-taking, social support-seeking, and self-development/self-advocacy happen in concert. This pattern is consistent with research that has defined proactive personality as a tendency to create opportunities by changing one’s environment instead of passively adapting to one’s environment (Bateman & Crant, 1993; Parker & Wang, 2015; Fuller et al., 2012). Participants’ focus on “communication and patience” and their propensity to “seek support from colleagues/parents” implies that proactivity is driven not only by individual agency, but also by resource-seeking and networking behaviours (Bell & Staw, 1990; Bakker et al., 2012). However, the data also shows that being proactive as an EFL candidate is interrelated with self-advocacy practices such as “knowing one’s rights and acting with a plan.” While this is supportive of conceptualizations linked to teacher agency (Borg, 2024; Farrell, 2018; Richards, 2015), the co-occurrence of these sub-dimensions in the pre-service sample is suggestive of a form of “readiness potential.” In the Turkish context, which is capable of bureaucratically centralization, these narratives may be the cognitive preparation of the candidates to assert their professional autonomy, even before they encounter actual constraints of the system.

Building on this proactive disposition, the results regarding job crafting (Table 4) indicate that the activities of relational and task crafting prevail; moreover, the strategies of “reducing demanding requests” and “cognitive reframing” are part of the repertoire of the participants. This pattern is consistent with the approach of re-shaping job design through employee initiative (Wrzesniewski & Dutton, 2001) and is consistent with the Job Demands-Resources framework (in which crafting increases job resources (Bakker et al., 2004)). The repetitive use of the term “building trust-based relationships with colleagues/parents” implies that relational crafting in the EFL context takes on a meaning by developing a sense of community. However, taking into account the hierarchic nature of the educational system, according to earlier studies (Gün, 2017; Khajavy et al., 2017; Pratt & Ashforth, 2003), the above results must be interpreted with a degree of reflexivity. The descriptions by the participants of highly supportive collaborative environments may reflect an “anticipatory crafting” strategy, which is an idealized projection of their future roles, rather than the complex reality of school cultures. This distinction brings up the possible gap between the “imagined community” of the candidates and the operational reality of the schools, implying the need for realistic negotiating strategies in teacher education.

Through these deliberate changes, the flow theme (Table 3) was revealed with four sub-dimensions: clear/unambiguous feedback, autotelic experience, transformation of time, and action-awareness merging. In particular, the triggering of flow by “immediate and clear signals from the student” (e.g., progress, “I get it!” moments) is consistent with the explicit goals and feedback conditions required by flow theory (Csikszentmihalyi, 2003; Bakker & van Woerkom, 2017). Considering that there is limited research that deals with flow among EFL teachers (Tardy & Snyder, 2004), the present findings provide an important contextual insight: classroom flow for novice teachers is concretized around “student-generated feedback.” This suggests that flow is not only a psychological reward, but a mechanism of validation of professional competence. Furthermore, the stories of “autotelic experience” and “action-awareness merging” mean that flow is related to sustainable professional enjoyment and sustained attention (Bakker, 2008), serving as a protective factor for the demands of the profession.

Finally, the work engagement dimension (Table 5) shows that dedication and vigour preside, sustained by intrinsic (passion for teaching, love of the profession) and extrinsic (recognition, seeing progress) elements. These findings are aligned with the literature in which engagement is defined as a state of high energy and identification with one’s work (Schaufeli et al., 2002; Hakanen et al., 2006) and the connection between engagement and resilience (Bakker & Bal, 2010; Diener, 2000; J. Han et al., 2016). However, in contrast to the studies showing “moderate levels” of engagement among practicing teachers in Turkey (Gün, 2017; Güçlü et al., 2014), the high engagement of the pre-service sample in this study signals a “career-entry optimism.” It is imperative to note that this vigour is currently fueled by intrinsic motivation and prospective hope. Therefore, in addition to confirming the psychological investment of the participants, these findings may also indicate that maintaining this high level of engagement will depend upon how these “anticipatory” resources interact with the actual job demands during the transition to the profession.

However, the interpretation of these findings should be approached cautiously. Given the small sample size and the specific institutional context of the Turkish EFL education system in which this study was conducted, the generalizability of these qualitative results may be limited. The proactive tendencies and high levels of engagement observed might be context-specific responses rather than universal traits. Therefore, there is a need for these findings to be replicated in diverse educational settings to confirm whether these anticipatory crafting strategies and flow experiences manifest similarly across different cultural and structural contexts.

## 6. Conclusions

This research was focused on the interrelationship of proactive personality, flow, job crafting, and engagement in professional development of pre-service EFL teachers in Turkey. The findings implicate proactive dispositions, manifested through initiative, help-seeking, and self-advocacy, on the one hand, and flow experiences, grounded in clear feedback and intrinsic enjoyment, on the other as the key drivers of relational and task crafting, and dedication and vigor. Taken together, these dynamics suggest that engagement is not the outcome of one factor, but the outcome of a virtuous cycle of proactive behaviors, meaningful interactions, and flow-conducive classroom conditions.

While limited in terms of its small sample size and context-specificity, the study does provide some very specific and actionable strategies for teacher preparation programs. In order to move beyond theoretical awareness, for example, teacher education curricula should explicitly incorporate “job crafting workshops” in which candidates are asked to redesign standard lesson plans so as to better align with their personal strengths and interests, and as a result develop task crafting skills. Furthermore, in order to foster the “unambiguous feedback” that is required for flow to occur, practicum components should make use of micro-teaching sessions with immediate and targeted debriefings as opposed to delayed assessments so that candidates can experience the direct relationship between action and result. Finally, to develop relational proactivity and self-advocacy, programs can use simulations of role-playing scenarios revolving around parent-teacher and colleague interactions, which can give candidates the confidence to mobilize social support networks before entering the profession.

At the same time, our present understanding of this process is still evolving. Much of the evidence is based on cross-sectional accounts which provide useful snapshots, but no complete picture of development. Future research, particularly longitudinal or intervention research, is needed to elucidate the development of pre-service teachers’ early expectations and “anticipatory crafting” strategies when faced with the actual structural realities of their first years of practice.

Ultimately, the research highlights that the creation of proactive, relationally grounded, and intrinsically rewarding professional practices may be at the core of sustaining teacher engagement. To reform teacher education in a way that contributes to the creation of resilient educators, curricula might need to shift from purely theoretical instruction to experiential, resilience-building models. By systematically providing candidates with concrete tools, such as structured reflective journals for cognitive reframing, peer-mentoring platforms to build relational capacity, and flexible lesson planning frameworks that allow for task modification, teacher educators can assist novices in actively developing their work. Furthermore, designing pedagogical environments that foster flow might involve incorporating extended practicum periods with reduced evaluative pressure, allowing pre-service teachers the autonomy to test interactive materials and receive immediate, formative student feedback. Through these structural reforms, the role of teacher education could be to contribute to the creation of educators who enter the profession not only with initial competence but with sustained confidence and commitment.

## Figures and Tables

**Table 1:** Socio-demographics of the Participants.

Participant	Gender	Age	Academic Year
P1	Female	20	2nd Year
P2	Female	21	3rd Year
P3	Female	21	3rd Year
P4	Female	21	3rd Year
P5	Female	20	2nd Year
P6	Female	21	3rd Year
P7	Male	21	3rd Year
P8	Female	20	2nd Year
P9	Female	21	3rd Year
P10	Female	21	3rd Year
P11	Female	22	4th Year
P12	Female	22	4th Year
P13	Female	21	3rd Year

**Table 2:** Thematic analysis of interview related to proactive personality.

Theme	Sub-Theme	Code	Sample Answers
Proactivity	Initiative in Problem Solving	Solution-oriented approach to overcome challenges	“I will approach challenges with a solution-oriented mindset …” (P1)
		Handling problems through communication and patience	“I believe that I can handle problems through communication and understanding. However, if I face a more serious issue, I think patience is the key to overcoming it.” (P9)
	Seeking and Utilizing Support	Proactively seeking support from colleagues and parents	“I will … seek support from colleagues or parents when needed …” (P2)
		Using a solution-focused mindset and seeking colleague support	“To handle challenges … approaching issues with a solution-focused mindset … and seeking support from colleagues when needed will be essential strategies for me.” (P11)
	Continuous Self-Improvement and Self-Advocacy	Structured planning and self-advocacy	“First and foremost, I will ensure that my lesson plans are well-structured to prevent any disruptions. Additionally, I will be aware of my rights as a teacher to handle any challenges effectively.” (P6)
		Continuous self-improvement through learning	“To overcome challenges … I aim to continuously improve myself. By reading, learning, and gaining experience …” (P10)

**Table 3:** Thematic analysis of interview related to flow.

Theme	Sub-Theme	Code	Sample Answers
Flow	Unambiguous Feedback	Student application of knowledge provides clear, immediate feedback	“I believe one of the most motivating things for a teacher is … witnessing students implementing what they have learned in their lives …” (P1)
		Student progress offers clear and motivating signals	“… Additionally, seeing students making progress in lessons and witnessing their improvement is very motivating.” (P2)
		A clear “I got it!” moment from a student acts as immediate, unmistakable feedback that fuels motivation	“For example, if I had a student who suddenly said, ‘I got it!’ and I saw that moment of realization in their eyes, that would bring me joy.” (P8)
	Autotelic Experience	Engaging, creative classroom activities that make learning enjoyable provide intrinsic fulfillment	“… Organizing creative activities and games in the classroom to make learning more enjoyable will bring me great joy.” (P10)
		Interactive activities and continuous self-improvement provide deep intrinsic rewards	“… Continuously improving myself and becoming a better guide for my students will be among my greatest sources of joy.” (P11)
		Intrinsic passion for teaching outweighs financial incentives	“Earning money and gaining financial independence can motivate a teacher at some point but teaching children with passion gives more motivation than money.” (P4)
	Time Transformation	Immersion in a topic causes a distorted perception of time, indicating deep flow	“The moments when a teacher immerses themselves in a topic, completely forgetting about time … are also highly motivating.” (P8)
	Action–Awareness Merging	Being fully immersed in the lesson creates a seamless merging of action and awareness	“… Being immersed in the natural flow of lessons allows teachers to engage seamlessly in activities and ensure smooth progression in the classroom.” (P13)

**Table 4:** Thematic analysis of interview related to job crafting.

Theme	Sub-Theme	Code	Sample Answers
Job Crafting	Relational Crafting	Developing deeper, trust-based relationships with students over time	“Over time, I hope to develop deeper relationships with students …” (P6)
		Engaging in open dialogue and collaboration with colleagues and parents	“I will constantly engage in dialogue and collaboration … and communicate openly with parents.” (P7)
		Building a supportive community among colleagues and fostering trust-based, communication with parents.	“As for colleagues, they can form a community that supports development through solidarity and sharing. Regarding parents, I believe establishing a trust-based and supportive communication is crucial.” (P8)
	Task Crafting	Modifying teaching methods to re-engage students	“When students are not engaged … The best way to overcome this is by changing teaching methods …” (P5)
		Adapting teaching methods and engaging in continuous professional development	“… Continuously developing oneself, and exploring new teaching methods are essential strategies.” (P13)
	Decreasing Hindering Job Demands	Implementing self-care and personal strategies to counter emotional exhaustion and reduce workload pressures.	“Emotional exhaustion, curriculum pressure … To overcome these, teachers should develop their own strategies … and take time for themselves.” (P8)
		Addressing time management and workload challenges to achieve a better work–life balance.	“… I think managing my time will be hard—lesson planning, grading, and maintaining a work–life balance will take time to get used to.” (P9)
		Implementing self-care and leveraging professional collaboration to mitigate burnout and bureaucratic obstacles.	“… Working in a supportive school environment … and implementing self-care strategies will allow teachers to remain strong and overcome bureaucratic obstacles.” (P11)
	Cognitive Crafting	Reframing challenges by cultivating patience, innovation, and a positive mindset to better manage classroom difficulties.	“Teachers face many challenges … they must remain patient and rational … by cultivating patience, many challenges can be managed effectively.” (P7)

**Table 5:** Thematic analysis of interview related to engagement.

Theme	Sub-Theme	Code	Sample Answers
Engagement	Dedication	Passion for education and making a difference	“Passion for education and a genuine desire to make a difference in students’ lives play a significant role.” (P3)
		Love for the profession and continuous self-improvement	“Loving the profession, maintaining motivation, and continuously improving oneself.” (P5)
		Commitment reinforced by recognition and impact	“When a teacher feels that their work is valuable and sees the impact of their efforts, they are more likely to continue their career for many years.” (P10)
	Vigor	Ever-changing student profile gives vitality	“The ever-changing student profile gives vitality to the teacher. The teacher tests his/her own teaching skills against the difficult student.” (P4)

## Data Availability

The datasets generated and analyzed during the current study are available from the corresponding author on reasonable request. None of the experiments were preregistered.
